# The ethical and legal challenges of human foetal brain tissue-derived organoids: At the intersection of science, ethics, and regulation

**DOI:** 10.1038/s44319-024-00099-5

**Published:** 2024-03-04

**Authors:** Tsutomu Sawai, Masanori Kataoka

**Affiliations:** 1 Graduate School of Humanities and Social Sciences, Hiroshima University, Higashi-Hiroshima, Japan; 2 Institute for the Advanced Study of Human Biology (ASHBi), Kyoto University, Kyoto, Japan; 3 Centre for Biomedical Ethics, Yong Loo Lin School of Medicine, National University of Singapore, Singapore, Singapore

**Keywords:** Development, Economics, Law & Politics, Neuroscience

## Abstract

The recent generation of brain organoids from human foetal tissue highlights the need for nuanced ethical considerations and international coordination to navigate the complexities of this research and its broader implications for developmental neuroscience and ethical debates.

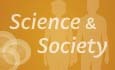

Earlier this year, Hendriks et al (2024) generated brain organoids (BOs) from human foetal tissue, which represents a substantial advancement in developmental neuroscience. Pluripotent stem-cell (PSC)-derived BOs have played a pivotal role in understanding the intricate early brain development processes but they have certain limits. In contrast, Hendriks et al (2024) reported that tissue stem-cell (TSC)-derived organoids accurately recapitulate the cellular characteristics of native human brain tissue and noted that “TSC-organoids can be established as robust, long-term expanding lines, while PSC-organoids typically progress toward a certain endpoint of the developmental trajectory.” Accordingly, PSC- and TSC-derived BOs offer complementary models for studying brain development.

Hendriks et al (2024) grew their organoids, which they designated as foetal brain organoids (FeBOs), from human foetal tissues at gestational weeks 12–15, obtained from elective abortions at a Dutch medical center and from an UK-based biobank. The analysis of FeBOs, along with other types of BOs, can potentially further advance our understanding of human brain development. This aspect is particularly significant in light of recent advancements in embryonic models that accurately simulate post-implantation morphology and execute key developmental processes, such as gastrulation and neurulation, which in turn lead to organogenesis (Amadei et al, 2022).

## Ethical, legal and social implications of FeBO research

FeBOs provide an accurate representation of foetal neural tissues at specific developmental stages and may actually help to reduce the use of foetal neural tissue in scientific research. However, initial and comparative studies still require growing BOs from foetal tissue. Consequently, research involving FeBOs finds itself at complex ethical crossroads, intertwining the moral quandaries associated with both BO research and the use of foetal tissues predominantly sourced through elective abortions from anonymous donors (Brumbaugh et al, 2023).

… research involving FeBOs finds itself at complex ethical crossroads, intertwining the moral quandaries associated with both BO research and the use of foetal tissues…

The ethical challenges associated with BO research (Sawai et al, 2022) apply to FeBO research as well. Dialogues among leading research organisations, including the US National Academies of Sciences, Engineering, and Medicine, and the International Society for Stem Cell Research (ISSCR), have already addressed these challenges (https://nap.nationalacademies.org/catalog/26078/; https://www.isscr.org/guidelines/). Central concerns are the potential that lab-grown human BOs might achieve consciousness and the ethical implications of transplanting them into animal models (de Jongh et al, 2022). The discourse also encompasses issues related to consent (Kataoka et al, 2024), commercialisation (Boers et al, 2016), integration with computational technologies (Kagan et al, 2023), and legal ramifications (Kataoka et al, 2023b). In addition, the public perception of BOs, which is often shaped by media representations, frequently diverges from scientific reality (Presley et al, 2022).

BO generation and their various applications, such as animal transplantation or integration with information technology, can evoke moral sensitivity among donors, which requires thorough informed consent. This issue is of even greater significance in the context of FeBOs, considering the complex challenges associated with foetal tissue acquisition. In addition, cell donors may feel a personal connection with their cell-derived BOs (Bollinger et al, 2021) and the sentiment of foetal tissue donors towards FeBOs are likely to be even more intricate. This demands more nuanced explanations than those in previous foetal tissue research.

Hendriks et al (2024) also acknowledge the intricate issue of consciousness. This controversial matter, previously confined to PSC-derived BOs, has become even more complex with FeBOs, as these can mature beyond the developmental stages of the original foetal tissues. The Hendriks et al study generated FeBOs from foetal brain tissue at gestational weeks 12–15, which underwent further maturation under modified culture conditions. This situation approaches the proposed ethical boundary in BO research, at the equivalence of the 20-week human brain (Koplin and Savulescu, 2019). Herein lies a dilemma: the increasing complexity of organoid development might inadvertently move them within broader bioethical debates.

This issue also extends to the domain of animal transplantation and integration with computational technology. Recent findings indicate that BOs, when transplanted into animals, tend to mature further within the host brain (Revah et al, 2022). However, the ramifications of in vivo maturation, particularly for FeBOs, and their impact on both organoids and host animals remain ambiguous and warrant scrutiny (Kataoka et al, 2023a).

Furthermore, FeBOs necessitate considering the regulatory discrepancy between embryo and BO research. The well-established ‘14-day rule’ for research on human embryos restricts in vitro cultivation to no more than 14 days after fertilisation or until the appearance of the primitive streak, whichever comes first. However, advances in embryology and biomedical sciences have sparked debates about the appropriateness of this rule. Although ISSCR has advocated for public discourse prior to any changes to the 14-day rule (https://www.isscr.org/guidelines/), existing guidelines generally prohibit the culture of human embryos beyond this period (Sawai et al, 2021). Generating BOs from 12–15-week-old foetal tissue therefore raises significant ethical questions.

If the ethical argument for BO research hinges on the underdevelopment of the nervous system, a parallel rationale may be applicable to embryonic research. Recent studies have shown that neurogenesis has not initiated in embryos at 14 days post-fertilisation (Tyser et al, 2021), highlighting the need for increased dialogue to clarify the ethical contours of BO versus embryonic research. In this regard, some have suggested that the 14-day rule should not be an absolute ethical boundary, but rather an intermediate constraint, and recommended its extension to 28 days (Appleby and Bredenoord, 2018). This is supported by the characterisation of the developmental period from 14 to 28 days as the “black box” of human ontogeny (Hurlbut et al, 2017).

## Regulations governing human foetal tissue research

Human foetal tissue research is marked by a notable absence of comprehensive international regulation, leading to a heterogeneous regulatory landscape with varying legal and ethical frameworks. This disparity stems from the distinct sociocultural, ethical and legal paradigms that shape each nation’s approach, resulting in a wide spectrum of practices and regulatory structures in this ethically sensitive and complex field (Yui et al, 2023).

Human foetal-tissue research is marked by a notable absence of comprehensive international regulation, leading to a heterogeneous regulatory landscape with varying legal and ethical frameworks.

In the USA, the use of human foetal tissue, particularly from elective abortions, is intricately intertwined with the polarising debate on abortion. This led to policy fluctuations under various administrations. The Trump administration implemented stringent restrictions on federal funding for human foetal tissue research, necessitating rigorous scientific justification and advanced informed consent procedures. Although measures have been relaxed under the Biden administration, stringent justifications and comprehensive procedural protocols for human foetal-tissue research remain intact.

The UK has been a forerunner in establishing guidelines for human foetal tissue research. Landmark frameworks such as the Peel Code in 1972 and the Polkinghorne Report in 1989 have concentrated on ethical dimensions, particularly regarding consent for tissue donation. Although not legally enforceable, these guidelines have profoundly influenced global standards, including the 1994 Guidelines of the Network of European CNS Transplantation and Restoration (NECTAR) and the 2021 Guideline of the ISSCR, emphasising both legal and ethical considerations, especially in the context of consent (Boer, 1994; https://www.isscr.org/guidelines/). These considerations also include separating the abortion decision-making process from considerations regarding human foetal tissue donation.

Japan has a more liberal stance towards abortion under the Maternal Protection Law, which permits abortion under specific circumstances. However, the regulatory framework governing the use of human foetal tissue in research, particularly for foetuses less than 12 weeks old, remains ambiguous and lacks comprehensive guidelines, including those pertaining to informed consent.

In the Netherlands, where the Hendriks et al study was conducted, the regulation of foetal tissue research is governed by the Fetal Tissue Act of 2001, which provides a comprehensive framework for the handling of embryos and foetuses in research. This act delineates specific guidelines for the timing and procedure of obtaining informed consent for the use of foetal tissues in research. It also established an implementation code, outlines the permissible objectives for the storage and use of foetal tissues, and requires informed consent from both parents.

In Germany, research on human embryos and foetal tissue is strictly regulated by law and ethical guidelines. The Embryo Protection Act of 1990 strictly forbids the use of surplus embryos for research and prohibits the creation of embryos for research or cloning. In addition, the German Medical Association (GMA) issued guidelines in 1991 permitting the use of aborted foetuses for research purposes, but in 1998 banned the use of foetal neural cells for the treatment of Parkinson’s disease due to ethical concerns. The German Research Foundation (DFG) also provides guidelines for stem-cell research, including foetal tissue, to ensure that research adheres to strict ethical standards.

While there is no explicit legal framework in Israel that specifically addresses human fetal tissue research, the country’s regulatory landscape for human embryonic stem cell (ESC) research and human cloning offers insights into its approach to bioethical governance. Israel’s permissive stance on ESC research, as highlighted by the Bioethics Advisory Committee of the Israeli Academy of Sciences and Humanities, reflects a broader ethics that does not ascribe full human dignity to pre-implantation embryos, thereby facilitating a more liberal research environment. The 1999 Law on the Prohibition of Genetic Intervention, while placing a moratorium on human cloning for reproductive purposes, does not directly regulate therapeutic cloning. Furthermore, the absence of specific regulation of emerging biotechnologies, such as brain organoids, suggests that Israeli policy may be characterized by a reactive rather than a preventive regulatory style, potentially adapting existing ethical frameworks to new scientific developments rather than creating new legislation for each innovation. This implies that ethical deliberation within the scientific community, taking into account the “14-day rule” and other established bioethical principles, will continue to play a central role in guiding research practices (Prainsack and Firestine, 2006; see also https://www.loc.gov/item/global-legal-monitor/2016-06-07/israel-law-prohibiting-human-cloning-amended/).

## Ethical and regulatory considerations in FeBO research

The expanding field of FeBO research holds substantial scientific and medical promises, particularly for complementing PSC-derived BOs. However, as delineated by Hendriks et al (2024), this introduces a set of ethical challenges, primarily concerning consent for the use of foetal tissue. Beyond these issues, their research underscores that FeBO studies may intensify ethical dilemmas in areas such as potential consciousness of BOs, animal transplantation and  integration of BOs with information technology. Moreover, it intersects with core ethical debates in embryology, notably the 14-day rule in embryo research, and the broader public discourse on abortion rights.

In the current landscape, there is a notable lack of comprehensive international and national regulatory frameworks specifically addressing FeBO research. If the premises articulated in this commentary are valid, research involving BOs, including FeBOs, may have to be subjected to stricter regulation, underpinned by sound ethical and legal foundations. The unique complexities and emerging ethical dilemmas of FeBO research require a sophisticated, globally coordinated regulatory approach. The importance of the 14-day rule in countries such as the UK and Japan underlines the need for in-depth national debates before changes are made that are compatible with foetal tissue research. However, this measure alone may prove insufficient, as there is a risk that some countries may prematurely declare that they are not bound by such regulations. In the meantime, funding agencies might also restrict funding for research in countries that take a less restricted approach to foetal tissue and BO-related research, potentially leading to the disintegration of the international research community. Consequently, the formulation of new, internationally accepted norms, while challenging, has become a critical requirement.

The unique complexities and emerging ethical dilemmas of FeBO research require a sophisticated, globally coordinated regulatory approach.

The importance of the 14-day rule in countries such as the UK and Japan underlines the need for in-depth national debates before changes are made that are compatible with foetal tissue research.

### Supplementary information

Peer Review File

## Supplementary information

A peer review file is available at 10.1038/s44319-024-00099-5.
