# Supplementary material for: The ethical and legal challenges of human foetal brain tissue-derived organoids: At the intersection of science, ethics, and regulation
Source: EMBO Rep. 2024 Mar 4;25(4):1700–3. doi: 10.1038/s44319-024-00099-5 (PMC11015037; doi:10.1038/s44319-024-00099-5)
Supplement: Supplementary file 1 — Peer Review File [file 44319_2024_99_MOESM1_ESM.pdf]

EMBO reports

At the Intersection of Science, Ethics, and Regulation

Tsutomu Sawai, Masanori Kataoka

Corresponding author(s): Tsutomu Sawai, (tstmsw@hiroshima-u.ac.jp)

Review Timeline:

Submission Date:	6th Feb 24
Accepted:	12th Feb 24

Editor: Holger Breithaupt

Transaction Report: This article was editorially reviewed.
